# Increase in Ocular Syphilis Cases at Ophthalmologic Reference Center, France, 2012–2015

**DOI:** 10.3201/eid2402.171167

**Published:** 2018-02

**Authors:** Ana Catarina Pratas, Pablo Goldschmidt, David Lebeaux, Claire Aguilar, Natalia Ermak, Jonathan Benesty, Caroline Charlier, Edgar Benveniste, Lilia Merabet, Neila Sedira, Emilie Hope-Rapp, Christine Chaumeil, Bahram Bodaghi, Emmanuel Héron, José-Alain Sahel, Olivier Lortholary, Marie-Hélène Errera

**Affiliations:** Quinze-Vingts National Eye Hospital, Paris, France (A.C. Pratas, P. Goldschmidt, N. Ermak, J. Benesty, E. Benveniste, L. Merabet, N. Sedira, C. Chaumeil, B. Bodaghi, E. Héron, J.-A. Sahel, M.-H. Errera);; DHU Sight Restore, Paris (A.C. Pratas, P. Goldschmidt, N. Ermak, J. Benesty, E. Benveniste, L. Merabet, N. Sedira, C. Chaumeil, B. Bodaghi, E. Héron, J.-A. Sahel, M.-H. Errera);; Sorbonne Universités, Université Pierre and Marie Curie, Université Paris, Paris (A.C. Pratas, P. Goldschmidt, N. Ermak, J. Benesty, E. Benveniste, L. Merabet, N. Sedira, C. Chaumeil, B. Bodaghi, E. Héron, J.-A. Sahel, M.-H. Errera);; Université Paris Descartes, Sorbonne Paris Cité, Assistance Publique–Hôpitaux de Paris, Necker-Enfants Malades Hospital, Necker-Pasteur Center for Infectious Diseases and Tropical Medicine, Paris (D. Lebeaux, C. Aguilar, C. Charlier, O. Lortholary);; Imagine Institute, Paris (D. Lebeaux, C. Aguilar, C. Charlier, O. Lortholary);; Centre Hospitalier Sainte-Musse, Toulon, France (E. Hope-Rapp); Pitié-Salpêtrière Hospital, Paris (B. Bodaghi)

**Keywords:** epidemiology, uveitis, neurosyphilis, ocular, syphilis, ophthalmologic, bacterial infections, men who have sex with men, MSM, sexually transmitted infections, France

## Abstract

We describe the frequency, demographic and clinical features, and visual outcomes of ocular syphilis infections observed during 2012–2015 at a tertiary reference center in Paris, France. Twenty-one cases (29 eyes) were identified. The occurrence of ocular syphilis increased from 1 case in 2012 to 5 cases in 2013, 6 cases in 2014, and 9 cases in 2015 (2.22–25.21/1,000 individual patients/year for the period). Among case-patients, an annual 20%–33% were co-infected with HIV. Seventy-six percent of ocular syphilis infections occurred in men who have sex with men. Seventy-five percent of case-patients had a good final visual outcome (best-corrected visual acuity >0.3 logMAR score). Visual outcome was worse for HIV-positive patients than for HIV-negative patients (p = 0.0139). At follow-up, the best visual outcomes were observed in patients whose mean time from first ocular symptom to consultation was 15 days (SD +19 days).

Syphilis is a sexually transmitted disease caused by the bacterium *Treponema pallidum*, which can infect almost any part of the body (*1*). Depending on the stage of the disease, acquired syphilis is classified into early (primary, secondary, and early latent) syphilis and late (or tertiary) syphilis (*2*,*3*). Although most cases of ocular syphilis occur in the context of tertiary syphilis, approximately one third of reported cases occur in the context of primary and secondary syphilis (*4*,*5*). Uveitis is observed in 0.6%–2.0% of patients with syphilis in any stage and up to 9% in patients co-infected with syphilis and HIV (HIV) (*6*–*10*). Syphilitic uveitis is considered a great mimicker because it can manifest as anterior uveitis, posterior uveitis, or panuveitis (*11*–*13*). Failure to recognize the ocular manifestations of syphilis or delayed treatment can lead to irreversible visual loss (*14*,*15*).

Syphilis has recently reemerged in the Western world in the context of the HIV crisis and massive sexual behavior changes among men who have sex with men (MSM). In 2015, nearly two thirds (62%) of the syphilis cases with information on transmission category were reported in MSM (*16*). 

In this context, we set up a retrospective study to review ocular syphilis cases observed during 2012–2015 in a tertiary reference center in Paris, France. Our objectives were to analyze trends in ocular syphilis frequency, demographic characteristics, clinical severity, and patient outcome.

## Methods

We reviewed the medical records of patients examined at the uveitis clinic (Centre Hospitalier National d’Ophtalmologie des Quinze-Vingts, Paris, France) from January 2012 through December 2015. We retrospectively identified patients with a new diagnosis of ocular syphilis. During the study period, 1,493 new patients were examined for uveitis. Patients meeting all the following criteria were included: age >18 years, having intraocular inflammation specifically affecting the uvea, and having a syphilis infection confirmed by the Venereal Disease Research Laboratory (VDRL) test. We defined a positive serologic test result as a positive *T. pallidum* hemagglutination assay (TPHA) and a positive VDRL test result in accordance with the classification of syphilis (*17*,*18*). We used a standardized strategy to diagnose uveitis (*19*), including anti-*Treponema* antibody detection in all patients in whom uveitis was diagnosed, with additional tests guided by the clinical context and paraclinical findings. 

We reviewed files to collect the following data: demographic information, including MSM status (in case of sexually transmitted disease, sexual orientation was recorded in the patient’s confidential file according to national ethics guidelines); duration of symptoms before diagnosis; and history of extraophthalmologic signs. Ophthalmologic examination included the measurement of the best-corrected visual acuity (BCVA) at initial presentation, at 15 days, and at the last follow-up. We converted BCVA to a visual acuity (VA) score on the basis of the logarithm of the minimum angle of resolution (logMAR).

We examined results from the slit lamp examination (in the anterior segment of the eye) and the fundus examination. We used the most informative imaging modality in patients with retinal inflammation, fundus fluorescein angiography, to report characteristic features of retinal vasculitis, optic nerve head inflammation, and macular edema. We used other fundus imaging studies such as indocyanine green angiography to assist in cases of choroidal inflammation, fundus autofluorescence imaging to assess retinal pigment epithelium integrity, and spectral-domain optical coherence tomography to evaluate retinal damage.

Applying the International Uveitis Study Group criteria, we classified uveitis into the following subtypes according to the site of inflammation: anterior uveitis, intermediate uveitis, posterior uveitis, or panuveitis (*20*,*21*). We considered placoid chorioretinitis to be the presence of >1 placoid, yellowish, outer retinal lesions. Fundus fluorescein angiography, indocyanine green angiography, and fundus autofluorescence indicate a typical fluorescent pattern in cases of placoid chorioretinitis (*22*,*23*).

Biological data included the results of treponemal and HIV serologic tests and the results of cerebrospinal fluid (CSF) analysis. The decision to perform lumbar puncture was left to the clinician’s discretion. We defined neurosyphilis as positive results in nontreponemal and treponemal serologic tests, combined with neurologic and CSF abnormalities, such as high leukocyte or protein concentrations (>0.5 g/L), and 1 CSF abnormality, such as positive VDRL or fluorescent treponemal antibody absorption test results (*24*–*26*) or a positive PCR test result for *T. pallidum*. Treatment and outcome data included the type, dose, duration, and route of antibiotic administration and corticosteroid treatment (local or systemic) as well as final VA.

We tested data variable distributions for normality and, when appropriate, we performed *t*-tests or equivalent nonparametric tests. We used 1-way analysis of variance when >3 groups were present and defined statistical significance as p<0.05. We analyzed the data by using R 3.2.2 software (https://cran.rproject.org/bin/windows/base/old/3.2.2).

## Results

**Figure 1:**
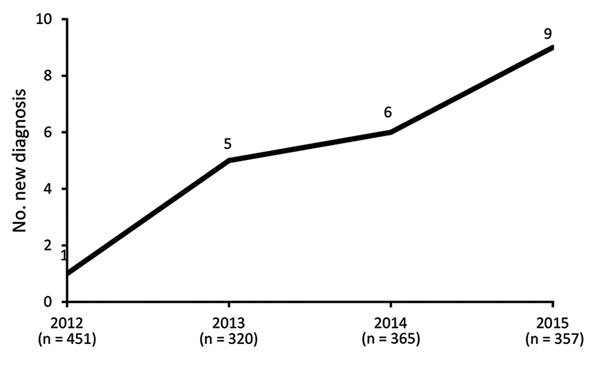
Number of newly diagnosed ocular syphilis cases among patients seen at a uveitis clinic, Paris, France, 2012–2015: 2012, 1 case; 2013, 5 cases; 2014, 6 cases; 2015, 9 cases.

We identified 21 cases of ocular syphilis (29 eyes) during January 2012–December 2015. Visits to outpatient clinics for confirmed ocular syphilis (the number of cases compared to the number of consultations for uveitis) increased from 1 in 2012 to 5 in 2013, 6 in 2014, and 9 in 2015 (2.22–25.21 visits/1,000 individual patients per year for the period Figure 1).

### Demographic Data, Biologic Data, and Clinical Features 

Twenty-one patients were affected, all men. The median age at presentation was 49 years (range 22–72 years). No difference in the mean age of patients was observed at diagnosis of ocular syphilis in 2013, 2014, and 2015. Median follow-up was 2 months (range 1–22 months).

**Figure 2:**
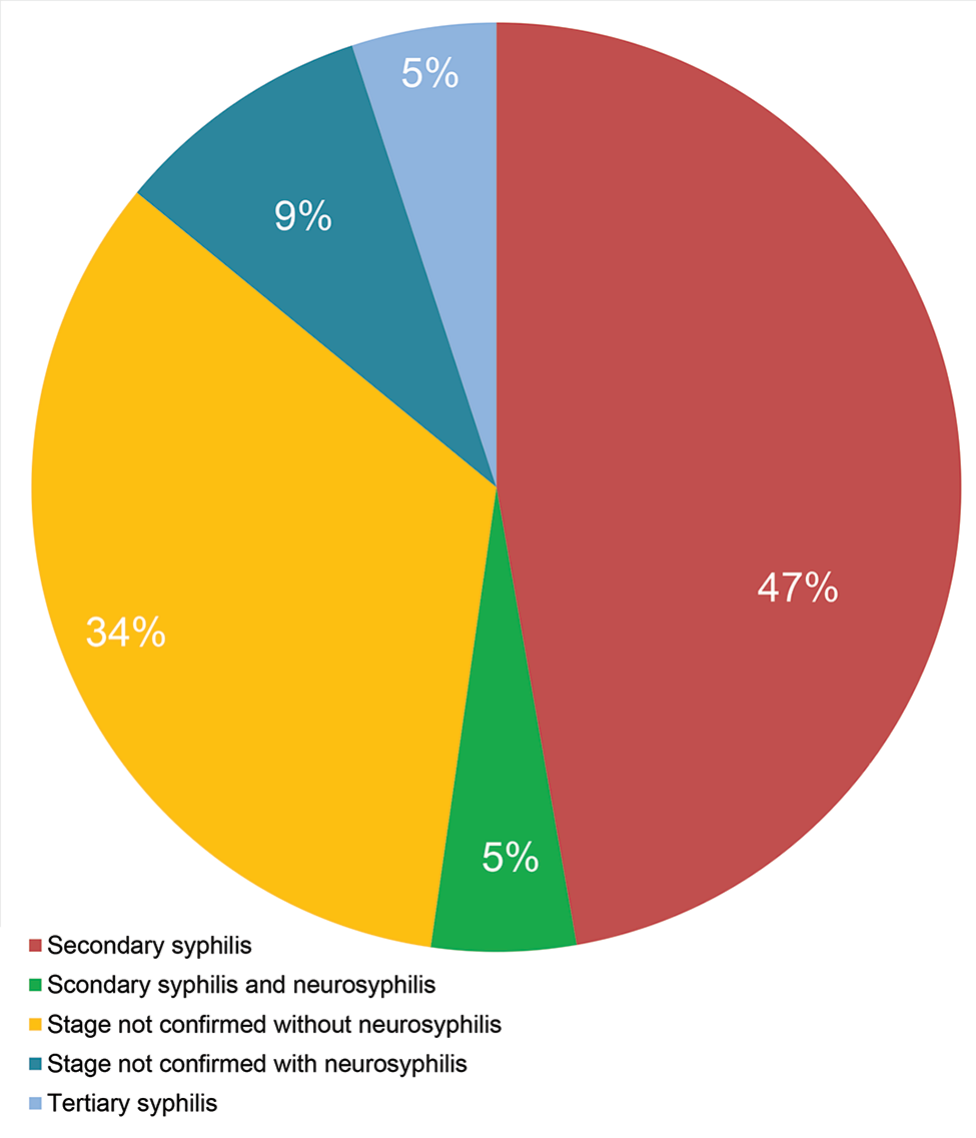
Presence of neurosyphilis and stages of syphilis in 21 patients with ocular syphilis seen at a uveitis clinic, Paris, France, 2012–2015.

**Figure 3:**
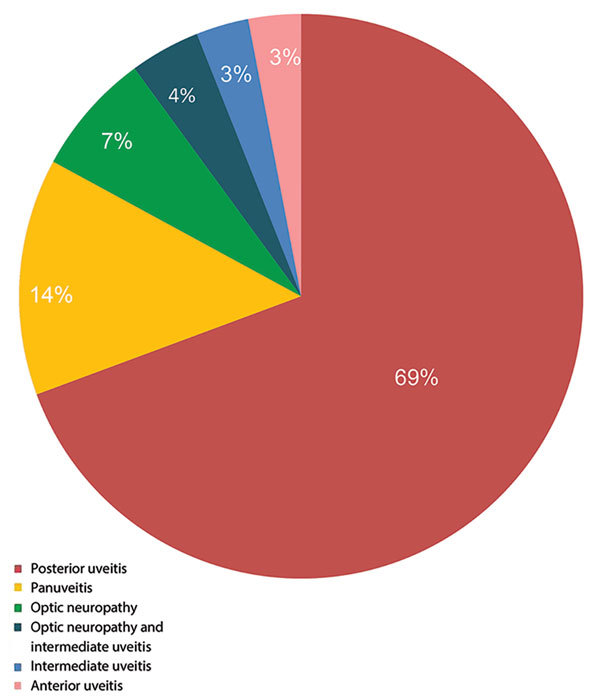
Anatomical sites of ocular inflammation in 21 patients with ocular syphilis seen at a uveitis clinic, Paris, France, 2012–2015.

At the time of uveitis diagnosis, 14 (67%) patients reported extraophthalmologic features, including rash, ulcer, mucocutaneous lesions, or neurologic signs. We noted the stages of syphilis and the presence of neurosyphilis in the 21 patients with ocular syphilis (Figure 2). Tertiary syphilis was diagnosed in only 1 (5%) patient, secondary syphilis in 11 (52%) patients, and an undetermined stage of syphilis in 9 (43%) patients. Of the 14 patients who had a lumbar puncture to assess CSF for evidence of neurosyphilis, 3 (21%) patients with ocular syphilis had neurosyphilis, and in 7 (50%) patients the CSF showed a lymphocytic reaction. We noted the frequency of different clinical presentations and anatomical types of ocular inflammation (Figure 3). The mean duration of ophthalmologic symptoms was 1 month before presentation at the uveitis clinic (range 1 day–4 months).

Isolated posterior uveitis was the most common type of uveitis observed within the cohort (69%), followed by panuveitis (14%). We summarized the frequency of clinical manifestations in patients with ocular syphilis (Table 1). Posterior placoid chorioretinitis was the most frequent finding in the posterior segment (58% of the total number of affected eyes); this condition accounted for 75% of cases of syphilis with eye involvement in 2013 and 81% in 2015.

**Table 1:** Clinical signs among patients with ocular syphilis seen at a uveitis clinic, Paris, France, 2012–2015

Clinical sign	No. (%) patients
Posterior placoid chorioretinitis	17 (58)
Retinitis	4 (14)
Optic neuritis	4 (14)
Anterior uveitis (plus iris gumma)	1 (3.5)
Neuroretinitis	1 (3. 5)
Intermediate uveitis	1 (3.5)
Retinal vasculitis	1 (3.5)

Patients with bilateral impairment had a mean BCVA of 0.9 logMAR (range 0–2.30), whereas patients with unilateral disease had a mean BCVA of 1.02 logMAR (p = 0.54). Worse final VA outcomes (BCVA >0.30 logMAR) were found in patients who waited a mean 61 days (SD +53 days) before seeking medical care. Patients who had the best final VA outcome (BCVA <0.30 logMAR) had waited a mean 15 days (SD +19 days) before seeking medical care.

### Effect of HIV Status

The results of HIV serologic tests were available for all patients. Six of the 21 patients (29%) were HIV-positive, and ocular syphilis led to the eventual diagnosis of HIV infection in 2 of these 6 cases. All but 1 of the 6 HIV-positive patients were MSM. In patients with ocular syphilis, HIV co-infection remained stable, ranging 20%–33% during 2012–2015. Most of the 21 patients were MSM, including 16 patients in the whole cohort and 5 of the 6 HIV-infected patients. These percentages did not vary over the study period. 

Subgroup analysis between HIV-negative and HIV-positive patients found no significant differences in the proportion of bilateral disease or initial VA. The clinical outcomes for affected eyes was significantly worse for HIV-positive patients (final mean BCVA 0.7 logMAR [range 0–2.3]) than for HIV-negative patients (0.09 logMAR [range 0–0.5]; p = 0.01) (Table 2). Affected eyes from HIV-positive patients had no significant difference in uveitis type compared with HIV-negative patients.

**Table 2:** Comparison of HIV-negative and HIV-positive patients with ocular syphilis seen at a uveitis clinic, by epidemiologic characteristics, clinical presentations, and outcomes, Paris, France, 2012–2015*

HIV status	HIV-negative patients, n = 15 patients, 22 eyes	HIV-positive patients, n = 6 patients, 7 eyes	p value
Median age (range), y	48 (32–72)	38.5 (22–61)	0.28
Median duration from onset of uveitis to presentation (range), d	7 (1–90)	30 (1–120)	0.98
Uveitis type, no. (% [of 29 eyes])			
Placoid chorioretinitis	15 (51)	2 (7)	
Retinitis	2 (7)	2 (7)	
Optic neuritis	3 (10.5)	1 (3.5)	
Anterior uveitis (plus iris gumma)	0	1 (3.5)	
Neuroretinitis	1 (3.5)	0	
Intermediate uveitis	1 (3.5)	0	
Retinal vasculitis	1 (3.5)	0	
Bilateral disease, no. (%)	7 (46)	1 (16.5)	
Neurosyphilis, no. (%)	4 (26)	6 (50)	
Mean initial visual acuity (range), logMAR score	0.9 (0–2.3)	1 (0–2)	0.61
BCVA (range) 2 weeks after starting treatment, logMAR score	0.5 (0–2.3)	1.2 (0.4–2)	0.0030
BCVA (range) at final follow-up, logMAR score	0.09 (0–0.5)	0.7 (0–2.3)	0.0139

*BCVA, best-corrected visual acuity; logMAR, logarithm of the minimum angle of resolution.

### Treatment

Most of the patients (17/21) were treated with daily intravenous penicillin, 3 patients received intravenous ceftriaxone, and 1 received oral doxycycline for 2 weeks followed by ceftriaxone for 2 weeks. The duration of treatment ranged from 2 to 3 weeks, although 2 patients received a total of 4 weeks of treatment (1 who was treated first with doxycycline and 1 who was diagnosed with tertiary syphilis). The penicillin dosage was 24 million IU/day for 13 patients, whereas 20 million IU/day was used in 4 patients (including 1 who was diagnosed with neurosyphilis). Five of the 7 patients with neurosyphilis were treated for 2 weeks.

In addition to receiving antibiotic therapy, 11 patients also received corticosteroid treatment orally (9 patients), periocular treatment (4 patients), or both (Technical Appendix Table 1). Corticosteroids were administered in 4 cases to treat an ocular Jarisch-Herxheimer reaction (JHR) (sudden onset of vitritis, papilledema, or both after initiation of systemic antibiotic therapy), and in the other 7 cases for persistence of ocular symptoms (papillitis, macular edema, or retinal vasculitis) despite appropriate antibiotic therapy. In 1 patient (patient 20), systemic corticosteroids were used to prevent systemic JHR (online Technical Appendix Table 2).

### Outcomes

**Figure 4:**
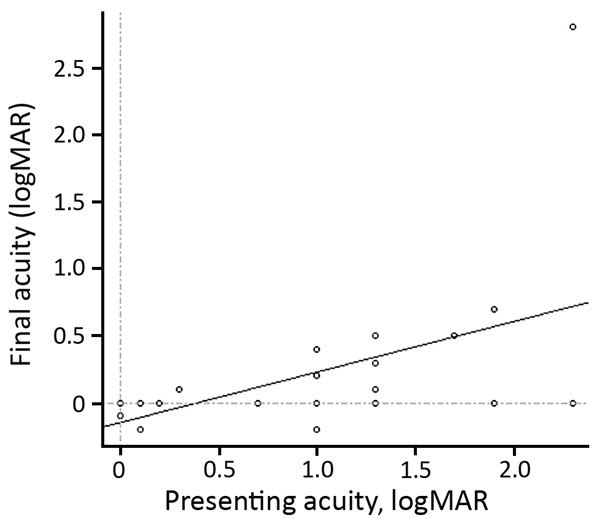
Visual acuity at initial examination versus final acuity (r = 0.5) in 21 patients with ocular syphilis seen at a uveitis clinic, Paris, France, 2012–2015.

Before antimicrobial drug therapy at initial presentation, 62% of patients had BCVA <0.3 logMAR (20/40 Snellen); after antimicrobial drug therapy at final follow-up, 75% had BCVA >0.3 logMAR (20/40 Snellen) (Figure 4). Of the 5 patients with a final BCVA >0.3 logMAR (20/40 Snellen), only 2 had a treatment delay (<12 weeks); therefore, we cannot correlate duration of treatment and visual prognosis because of the small number of patients with a poor outcome.

Most patients had better final VA than at presentation, with a mean initial BCVA of 0.9 logMAR (range 0–2.3) to a mean final BCVA of 0.2 logMAR (range –0.2 to 2.3). BCVA at presentation did not predict BCVA posttreatment (r = 0.5) (Figure 4). All patients had a good outcome in terms of quiescence of uveitis and final BCVA regardless of whether or not they had been treated with systemic or subconjunctival corticosteroids. No statistically significant difference in final BCVA after treatment was found between patients on the basis of whether or not they received systemic or subconjunctival corticosteroids: 0.40 logMAR (range 0–2.3) versus 0.15 logMAR (range –0.2 to 0.5), respectively (p = 0.208). Initial BCVA did not significantly differ between patients who had and those who had not received systemic or subconjunctival corticosteroids (p = 0.627).

In all patients who had lumbar puncture, no statistical difference was observed in final visual outcome between eyes in which a CSF lymphocytic reaction was diagnosed (final BCVA 0.46 [range 0–0.5] logMAR) and eyes with no evidence of CSF abnormalities (0.23 [range –0.2 to 2.3] logMAR). We also studied differences in syphilis trends regarding visual outcomes in 2013, 2014, and 2015. We observed no significant differences in visual recovery over time (p = 0.5783). In 13 patients for whom >3 months of follow-up was available for laboratory testing, quantitative VDRL screening was reported to decrease, except in 1 patient who had recurrence of uveitic syphilis because of an interruption in antiretroviral therapy (online Technical Appendix Table 2).

## Discussion

Our study examined the trends in ocular syphilis in patients attending the uveitis clinic in a tertiary uveitis center in France. During the 4-year study period, consistent 10-fold increase in ocular syphilis was observed. This finding is in agreement with an annual epidemiologic report of the European Centre for Disease Prevention and Control (ECDC), which observed that in some countries in Europe, including France, the United Kingdom, and Germany, a >50% increase in syphilis cases occurred during 2010–2014 (*16*). In France during 2012–2015, the number of early syphilis cases increased from nearly 750 to 1,450 reported cases. This trend was dramatically sharp among MSM (*27*). Indeed, a 15-fold increase was also found in the incidence of ocular syphilis in the United Kingdom during 1998–2003 (*15*).

A predominantly male cohort of patients has been reported in all studies, including ours (*7*,*14*,*22*,*23*,*28*). In our study, 76% of ocular syphilitic cases occurred in MSM. The proportion of HIV-positive MSM remained stable during the period among ocular syphilis case-patients. A recent report has shown that MSM account for the greatest increases in syphilis cases (*29*). In our study, most case-patients had syphilis diagnosed during the second stage of the disease.

Syphilis is known to display diverse ophthalmologic manifestations, which our study affirmed. The most common finding was posterior uveitis manifesting as placoid chorioretinitis (58%), which has been noted in previous reports (*14*,*23*,*29*–*31*). Posterior placoid chorioretinitis is an extremely specific clinical sign for the diagnosis of syphilis. Therefore, ≈60% of the patients had clinical features consistent with the diagnosis of syphilis on retinal imaging even before the diagnosis of systemic syphilis was established. Syphilis can be highly suspected by the ophthalmologist and requires confirmation with blood testing. Moreover, to detect other ocular presentations, blood testing for syphilis should be included in routine laboratory testing for all patients with ocular inflammation. However, clinical presentations of other conditions also were identified (anterior uveitis, intermediate uveitis, retinitis, and optic nerve edema). Notably, only 20% of patients had an inflamed anterior segment.

We found disparities in ocular syphilis trends by clinical phenotype and evidence of neurosyphilis on CSF analysis during the 4-year study period. By clinical feature, the greatest number of cases of posterior placoid chorioretinitis occurred in 2013 (4 patients) and 2015 (7 patients) and the least in 2014 (1 patient). Meanwhile, among the patients who underwent a lumbar puncture, 75% of those with ocular syphilis had a CSF lymphocytic reaction in 2013 (3/4 patients), which decreased to 33% in 2014 (2/6 patients) and showed an increase again to 50% in 2015 (2/4 patients). These differences in ocular syphilitic forms could be related to several treponemal strains originating from distinct regions and diverse isolation dates (*32*). Five different strains in 14 patients have been identified previously (*33*).

According to current guidelines, the recommended regimen for neurosyphilis and ocular syphilis is benzyl penicillin, 18–24 million units/day by intravenous injection for 10–14 days (*2**,**3*). In our study, this regimen proved to be effective. The duration of treatment was similar whether or not lumbar puncture confirmed neurosyphilis. Current guidelines recommend CSF examination only if patients have clinical evidence of neurologic involvement or ophthalmic symptoms.

In our study, of 14 case-patients who had undergone CSF analysis, 7 (50%) had a lymphocytic reaction and 3 (21%) had neurosyphilis based on a positive reactive treponemal or nontreponemal test or PCR test on CSF samples. These numbers are consistent with a previous report of 26% of 31 ocular syphilis case-patients having neurosyphilis diagnosed on CSF examination. In that study, neurosyphilis was diagnosed if CSF fluorescent treponemal antibody absorption test was reactive or if a lymphocytic pleocytosis of >20 cells/μL was detected (*34*). In contrast, the US Centers for Disease Control and Prevention’s estimate of 50%–75% of ocular syphilis patients with evidence of neurosyphilis based on a positive CSF VDRL result (*35*) is higher than in our study.

In our study, CSF results did not influence the duration of treatment or the outcome. Furthermore, the sensitivity of PCR tests on CSF could be questioned because sensitivity was low even in intraocular samples. Indeed, because of the variable sensitivity of CSF tests (*26*), no CSF test result can definitively exclude a diagnosis of neurosyphilis (*33*). Because experience with PCR tests on intraocular fluids is limited (*36*,*37*), we suggest not relying on CSF results in such complex cases, preferring to examine CSF only when an alternate or concomitant infection is suspected (e.g., *Herpesviridae* and *Mycobacterium tuberculosis* infections), because syphilis is known to mimic many other causes of uveitis. Recently, PCR in CSF has been shown to be highly specific in the diagnosis of neurosyphilis (*26*), offering guidance in case of treatment failure or in supporting the clinical diagnosis of ocular syphilis or neurosyphilis when several co-infections other than syphilis are also detected. Therefore, ocular syphilis should be considered in any patient with positive syphilis serologic tests and ophthalmologic findings consistent with ocular syphilis and should be treated with a regimen of penicillin appropriate for neurosyphilis regardless of CSF results.

This study has shown that VA on initial examination did not predict the final visual outcome because no correlation existed between BCVA at presentation and posttreatment. However, a longer delay in consulting for visual symptoms was associated with worse final VA outcomes. We found the best final outcomes (BCVA <0.30 logMAR; >6/12 Snellen) in patients whose mean time from the first visual symptom to consultation was 15 days (SD ±19) and, conversely, the worst final BCVA (>0.30 logMAR; <6/12 Snellen) in patients whose mean time from onset to consultation for visual symptoms was 2 months (SD +53 days). Similarly, a longer duration between the first presentation of uveitis and treatment for syphilis has been associated previously with a significantly higher logMAR VA (e.g., 12 weeks in a study by Bollemeijer et al. [*31*]).

The use of corticosteroids to control the degree of inflammation remains undefined in the current treatment guidelines. Oral and topical corticosteroids have been used in the past in association with antibiotic treatment (*4*,*23*,*38*–*40*). Although topical corticosteroids can be used freely to help control anterior segment inflammation (*40*), intravitreal injections of triamcinolone appear to be harmful (*39*,*41*). Previous studies highlight the usefulness of systemic corticosteroids (intravenous and oral) and periocular injections to treat macular edema (*4*), papillitis (*23*), and posterior placoid chorioretinopathy (*40*). The results of our study suggest that the use of periocular or oral steroids associated with antibiotic treatment could be useful as adjunctive therapy for ocular syphilis in the treatment of ocular JHR (vitritis), papillitis, and retinal vasculitis. Recently, Bollemeijer et al. found no difference in visual outcome whether or not patients had received any adjunctive systemic or local steroids; however, they indicate that adjunct corticosteroid treatment might have been preferable in the most severe cases (*31*).

If cases of persisting inflammation despite appropriate antibiotic therapy or an ocular JHR, corticosteroids seemed to be effective. In our study, improvements in VA after the use of periocular injection or systemic corticosteroids were observed in all 11 patients, independent of the route of corticosteroid administration (intravenous, oral, or subconjunctival). No difference in final VA after treatment was found between patients who had or had not received any adjunctive treatment with systemic or periocular corticosteroids.

Discrepancies exist in previous reports of HIV co-infection and visual outcome in syphilitic uveitis. In fact, in previous studies, HIV positivity has been associated with a worse visual outcome in cases of syphilitic uveitis. Other recent studies have not supported this conclusion (*1*,*7*,*30*,*42*–*45*). Bollemeijer et al. hypothesized that favorable outcomes might be attributable to the immune status of HIV-positive patients receiving highly active antiretroviral therapy (*31*). In our study, 3 of the HIV-positive patients had CD4+ cell counts <350 cells/µL, which might explain the worse visual outcomes associated with HIV co-infection. We report worse posttreatment VA in HIV-positive patients compared with HIV-negative patients (Table 2).

The main limitations of this study stem from its intrinsic retrospective nature, leading to a heterogeneous follow-up schedule for ophthalmic examination. We highlight the same limitation as noted in previous case series (the study was conducted in 1 uveitis center from referrals, so this population might not represent the total spectrum of syphilitic uveitis) (*31*). Nonetheless, we strongly believe that the study provides a thorough description of the cases of ocular syphilis diagnosed at a reference national eye center. This report is strengthened by the fact that we have epidemiologic information on case-patients, including the sex of their sex partners and HIV status, as well as ophthalmologic examination and visual outcome information.

Syphilis can involve visual function, and therefore clinicians dealing with patients at risk for syphilis should consider that uveitis is one of its potential indications. Despite the historic stigma of syphilis and the generally low prevalence in the population at large, *T. pallidum* screening of patients with uveitis should be conducted (*45*). The nonspecific symptoms of uveitis include severe redness, pain, floaters, photophobia, and blurred vision. All patients with ocular syphilis should undergo HIV testing and comprehensive counseling on the prevention of sexually transmitted diseases. A minimal decline has been observed in the number of HIV diagnoses per 100,000 population over the past decade. However, the trend by transmission mode shows that the number of HIV diagnoses among MSM has continued to increase in countries in Europe (*46*). These trends in HIV infection are consistent with the increasing incidence of syphilis infection among MSM. At this juncture, the availability of oral HIV preexposure prophylaxis and persons’ decisions to rely on it rather than condoms might require future evaluation of an increased risk for ocular impairment attributable to increasing numbers of syphilis infection.

Technical Appendix Clinical findings and steroid regime in patients requiring additional steroid treatment and biologic results, clinical findings, immune status, and treatment regimen in 21 patients with ocular syphilis seen at a uveitis clinic, Paris, France, 2012–2015.

## References

[1] Moradi A, Salek S, Daniel E, Gangaputra S, Ostheimer TA, Burkholder BM, et al. Clinical features and incidence rates of ocular complications in patients with ocular syphilis. Am J Ophthalmol. 2015;159:334–43.e1. 10.1016/j.ajo.2014.10.030 25447116

[2] Janier M, Hegyi V, Dupin N, Unemo M, Tiplica G, Potočni KM, et al. 2014 European guideline in the management of syphilis [cited 2016 Mar 13]. http://www.iusti.org/regions/europe/pdf/2014/2014SyphilisguidelineEuropean.pdf 10.1111/jdv.12734 25348878

[3] World Health Organization. Guidelines for the management of sexually transmitted infections [cited 2016 Apr 23]. http://apps.who.int/iris/bitstream/10665/42782/1/9241546263_eng.pdf?ua=1

[4] Davis JL. Ocular syphilis. Curr Opin Ophthalmol. 2014;25:513–8. 10.1097/ICU.0000000000000099 25237932

[5] Biotti D, Bidot S, Mahy S, Buisson M, Duong M, Grappin M, et al. Ocular syphilis and HIV infection. Sex Transm Dis. 2010;37:41–3. 10.1097/OLQ.0b013e3181b3e4d8 20118676

[6] Balba GP, Kumar PN, James AN, Malani A, Palestine AG, Welch JN, et al. Ocular syphilis in HIV-positive patients receiving highly active antiretroviral therapy. Am J Med. 2006;119:448.e21–5. 10.1016/j.amjmed.2005.11.016 16651059

[7] Mathew RG, Goh BT, Westcott MC. British Ocular Syphilis Study (BOSS): 2-year national surveillance study of intraocular inflammation secondary to ocular syphilis. Invest Ophthalmol Vis Sci. 2014;55:5394–400. 10.1167/iovs.14-14559 24925878

[8] Kiss S, Damico FM, Young LH. Ocular manifestations and treatment of syphilis. Semin Ophthalmol. 2005;20:161–7. 10.1080/08820530500232092 16282150

[9] Tamesis RR, Foster CS. Ocular syphilis. Ophthalmology. 1990;97:1281–7. 10.1016/S0161-6420(90)32419-3 2243678

[10] Aldave AJ, King JA, Cunningham ET Jr. Ocular syphilis. Curr Opin Ophthalmol. 2001;12:433–41. 10.1097/00055735-200112000-00008 11734683

[11] Bonnin N, Laurichesse H, Beytout J, Mrozek N, Lesens O, André M, et al. Ophthalmologists play a key role in the management of syphilis presenting with ocular involvement. Acta Ophthalmol. 2014;92:e328–9. 10.1111/aos.12315 24330492

[12] Anshu A, Cheng CL, Chee S-P. Syphilitic uveitis: an Asian perspective. Br J Ophthalmol. 2008;92:594–7. 10.1136/bjo.2007.133843 18441168

[13] Peeling RW, Hook EW III. The pathogenesis of syphilis: the Great Mimicker, revisited. J Pathol. 2006;208:224–32. 10.1002/path.1903 16362988

[14] Yang P, Zhang N, Li F, Chen Y, Kijlstra A. Ocular manifestations of syphilitic uveitis in Chinese patients. Retina. 2012;32:1906–14. 10.1097/IAE.0b013e3182509796 22495332

[15] Doris JP, Saha K, Jones NP, Sukthankar A. Ocular syphilis: the new epidemic. Eye (Lond). 2006;20:703–5. 10.1038/sj.eye.6701954 15933744

[16] European Centre for Disease Prevention and Control. Syphilis–annual epidemiological report 2015 [cited 2016 May 13]. https://ecdc.europa.eu/en/publications-data/syphilis-annual-epidemiological-report-2015

[17] Basse-Guérineau A-L, Dupin N, Ebel A, El Ghouzi M-H, Janier M. Diagnostic serologique de la syphilis [cited 2016 Mar 13]. http://www.invs.sante.fr/publications/2004/diag_sero_syphilis_230604/diag_sero_syphilis.pdf

[18] Haute Autorité de Santé. Modification de la nomenclature des actes de biologie médicale pour les actes de recherche du *Treponema pallidum* (bactérie responsable de la syphilis) [cited 2016 Jun 3]. http://www.has-sante.fr/portail/upload/docs/application/pdf/2015-05/argumentaire_syphilis_vd.pdf

[19] de Parisot A, Kodjikian L, Errera MH, Sedira N, Heron E, Pérard L, et al.; ULISSE group. Randomized controlled trial evaluating a standardized strategy for uveitis etiologic diagnosis (ULISSE). Am J Ophthalmol. 2017;178:176–85. 10.1016/j.ajo.2017.03.029 28366648

[20] Jabs DA, Nussenblatt RB, Rosenbaum JT; Standardization of Uveitis Nomenclature (SUN) Working Group. Standardization of uveitis nomenclature for reporting clinical data. Results of the First International Workshop. Am J Ophthalmol. 2005;140:509–16. 10.1016/j.ajo.2005.03.057 16196117 PMC8935739

[21] Matsumoto Y, Spaide RF. Autofluorescence imaging of acute syphilitic posterior placoid chorioretinitis. Retin Cases Brief Rep. 2007;1:123–7. 10.1097/01.iae.0000242759.80833.39 25390772

[22] Yap SC, Tan YL, Chio MTW, Teoh SC. Syphilitic uveitis in a Singaporean population. Ocul Immunol Inflamm. 2014;22:9–14. 10.3109/09273948.2013.829106 24063580

[23] Sahin O, Ziaei A. Clinical and laboratory characteristics of ocular syphilis, co-infection, and therapy response. Clin Ophthalmol. 2015;10:13–28. 10.2147/OPTH.S94376 26730177 PMC4694667

[24] Hay PE, Clarke JR, Taylor-Robinson D, Goldmeier D. Detection of treponemal DNA in the CSF of patients with syphilis and HIV infection using the polymerase chain reaction. Genitourin Med. 1990;66:428–32. 2265840 10.1136/sti.66.6.428 PMC1194582

[25] Noordhoek GT, Wolters EC, de Jonge ME, van Embden JD. Detection by polymerase chain reaction of *Treponema pallidum* DNA in cerebrospinal fluid from neurosyphilis patients before and after antibiotic treatment. J Clin Microbiol. 1991;29:1976–84. 1774324 10.1128/jcm.29.9.1976-1984.1991 PMC270245

[26] Vanhaecke C, Grange P, Benhaddou N, Blanche P, Salmon D, Parize P, et al.; Neurosyphilis Network; Neurosyphilis network. Clinical and biological characteristics of 40 patients with neurosyphilis and evaluation of *Treponema pallidum* nested polymerase chain reaction in cerebrospinal fluid samples. Clin Infect Dis. 2016;63:1180–6. 27585981 10.1093/cid/ciw499

[27] Ndeikoundam N, Viriot D, Fournet N, De Barbeyrac B, Goubard A, Dupin N, et al. Les infections sexuellement transmissibles bactériennes en France: situation en 2015 et évolutions récentes. Bull Epidemiol Hebd (Paris). 2016;41–42:738–44.

[28] Abara WE, Hess KL, Neblett Fanfair R, Bernstein KT, Paz-Bailey G. Syphilis trends among men who have sex with men in the United States and Western Europe: a systematic review of trend studies published between 2004 and 2015. PLoS One. 2016;11:e0159309. 10.1371/journal.pone.0159309 27447943 PMC4957774

[29] Tsan GL, Amin P, Sullivan-Mee M. Nongranulomatous Uveitis as the first manifestation of syphilis. Optom Vis Sci. 2016;93:647–51. 10.1097/OPX.0000000000000838 26927522

[30] Puech C, Gennai S, Pavese P, Pelloux I, Maurin M, Romanet J-P, et al. Ocular manifestations of syphilis: recent cases over a 2.5-year period. Graefes Arch Clin Exp Ophthalmol. 2010;248:1623–9. 10.1007/s00417-010-1481-z 20703496

[31] Bollemeijer JG, Wieringa WG, Missotten TOAR, Meenken I, ten Dam-van Loon NH, Rothova A, et al. Clinical manifestations and outcome of syphilitic uveitis. Invest Ophthalmol Vis Sci. 2016;57:404–11. 10.1167/iovs.15-17906 26848879

[32] Lhoir S, Willermain F, Jansen J, Libois A, Van Calster J, Caspers L. et al. Can we consider syphilitic uveitis as neurosyphilis? A retrospective analysis of lumbar puncture results in a cohort of syphilitic uveitis patients. Acta Ophthalmologica. 2013;91:0.

[33] Reekie I, Reddy Y. Use of lumbar punctures in the management of ocular syphilis. Semin Ophthalmol. 2016;•••:1–4; Epub ahead of print. 10.1080/08820538.2016.1228986 27860537

[34] Centurion-Lara A, Molini BJ, Godornes C, Sun E, Hevner K, Van Voorhis WC, et al. Molecular differentiation of *Treponema pallidum* subspecies. J Clin Microbiol. 2006;44:3377–80. 10.1128/JCM.00784-06 16954278 PMC1594706

[35] Oliver S, Sahi SK, Tantalo LC, Godornes C, Neblett Fanfair R, Markowitz LE, et al. Molecular typing of *Treponema pallidum* in ocular syphilis. Sex Transm Dis. 2016;43:524–7. 10.1097/OLQ.0000000000000478 27419819 PMC5253705

[36] Troutbeck R, Chhabra R, Jones NP. Polymerase chain reaction testing of vitreous in atypical ocular syphilis. Ocul Immunol Inflamm. 2013;21:227–30. 10.3109/09273948.2013.770887 23617827

[37] Booth J, Rodger A, Singh J, Alexander S, Hopkins S. Syphilitic panuveitis with retinal necrosis in an HIV positive man confirmed by *Treponema pallidum* PCR. J Infect. 2009;59:373–5. 10.1016/j.jinf.2009.08.022 19766672

[38] US Centers for Disease Control and Prevention]. 2015 sexually transmitted diseases treatment guidelines: syphilis [cited 2017 Feb 24]. Available at https://www.cdc.gov/std/tg2015/syphilis.htm

[39] Eandi CM, Neri P, Adelman RA, Yannuzzi LA, Cunningham ET Jr; International Syphilis Study Group. Acute syphilitic posterior placoid chorioretinitis: report of a case series and comprehensive review of the literature. Retina. 2012;32:1915–41. 10.1097/IAE.0b013e31825f3851 22863970

[40] Erol N, Topbas S. Acute syphilitic posterior placoid chorioretinitis after an intravitreal triamcinolone acetonide injection. Acta Ophthalmol Scand. 2006;84:435. 10.1111/j.1600-0420.2005.00641.x 16704715

[41] Pichi F, Ciardella AP, Cunningham ET Jr, Morara M, Veronese C, Jumper JM, et al. Spectral domain optical coherence tomography findings in patients with acute syphilitic posterior placoid chorioretinopathy. Retina. 2014;34:373–84. 10.1097/IAE.0b013e3182993f11 23860561

[42] Erol N, Topba S. Complications of intravitreal triamcinolone acetonide. Surv Ophthalmol. 2009;54:427, author reply 427–8. 10.1016/j.survophthal.2009.02.012 19422973

[43] Gass JD, Braunstein RA, Chenoweth RG. Acute syphilitic posterior placoid chorioretinitis. Ophthalmology. 1990;97:1288–97. 10.1016/S0161-6420(90)32418-1 2243679

[44] Amaratunge BC, Camuglia JE, Hall AJ. Syphilitic uveitis: a review of clinical manifestations and treatment outcomes of syphilitic uveitis in human immunodeficiency virus-positive and negative patients. Clin Experiment Ophthalmol. 2010;38:68–74. 10.1111/j.1442-9071.2010.02203.x 20447104

[45] Tucker JD, Li JZ, Robbins GK, Davis BT, Lobo A-M, Kunkel J, et al. Ocular syphilis among HIV-infected patients: a systematic analysis of the literature. Sex Transm Infect. 2011;87:4–8. 10.1136/sti.2010.043042 20798396 PMC3103105

[46] European Centre for Disease Prevention and Control/World Health Organization Regional Office for Europe. HIV/AIDS surveillance in Europe, 2015 [cited 2016 May 13]. https://ecdc.europa.eu/sites/portal/files/media/en/publications/Publications/HIV-AIDS-surveillance-Europe-2015.pdf

